# Microstructures define melting of molybdenum at high pressures

**DOI:** 10.1038/ncomms14562

**Published:** 2017-03-01

**Authors:** Rostislav Hrubiak, Yue Meng, Guoyin Shen

**Affiliations:** 1 High Pressure Collaborative Access Team (HPCAT), Geophysical Laboratory, Carnegie Institution of Washington, Argonne, Illinois 60439, USA

## Abstract

High-pressure melting anchors the phase diagram of a material, revealing the effect of pressure on the breakdown of the ordering of atoms in the solid. An important case is molybdenum, which has long been speculated to undergo an exceptionally steep increase in melting temperature when compressed. On the other hand, previous experiments showed nearly constant melting temperature as a function of pressure, in large discrepancy with theoretical expectations. Here we report a high-slope melting curve in molybdenum by synchrotron X-ray diffraction analysis of crystalline microstructures, generated by heating and subsequently rapidly quenching samples in a laser-heated diamond anvil cell. Distinct microstructural changes, observed at pressures up to 130 gigapascals, appear exclusively after melting, thus offering a reliable melting criterion. In addition, our study reveals a previously unsuspected transition in molybdenum at high pressure and high temperature, which yields highly textured body-centred cubic nanograins above a transition temperature.

## Abstract (web summary)

Molybdenum has long been speculated to undergo an exceptionally steep increase in melting temperature when compressed but without direct experimental evidence. Here authors claim such a direct observation and also report a transition at high pressure and high temperature.

There is a resurgence of interest in melting studies of molybdenum (Mo) because of the alarmingly large discrepancies in high-pressure melting temperature determination in literature^1^. Theoretical studies have predicted rapid increases of melting temperature in Mo with compression; at a pressure of 100 gigapascals (GPa), the majority of theoretical predictions^2^,^3^,^4^,^5^,^6^ and acoustic velocity measurements under shock compression^7^,^8^,^9^ indicate melting temperatures nearly 3,000 K higher than those obtained by all of the previous static compression experiments^10^,^11^,^12^,^13^. This discrepancy is highly dissatisfactory and imposes intolerable limitations on the validation of theoretical models of high-temperature transition metal melting in general^1^. Despite the newest experimental improvements^13^ and several hypotheses proposed to explain the apparent disagreement in the high-pressure melting of Mo (refs 12, 14, 15), reconciliation of the most recent reports is still unattained.

In static compression experiments, the laser-heated diamond anvil cell^16^ (LH DAC) is often used for generating simultaneously high pressure and temperature (*P*–*T*) conditions up to several hundreds of GPa and several thousands of degrees. The determination of melting is ultimately defined by the melting detection criterion used and by the temperature measured during the experiment. While the accuracy of temperature measurement method, that is, spectroradiometry, in LH DAC experiments still poses a challenge due to uncertainties in the material's emissivity and thermal gradients^17^, the disagreements in literature on the melting points of Mo at high pressure are simply too large to be accounted for by the uncertainties in temperature measurement. There are several other experimental factors, which may affect reliable melting determination, including large temperature variation during melting, chemical reactions and melt containment. These can be minimized by constraining the total heating duration in the LH DAC to tens of milliseconds, that is, using square-modulated pulse (SMP) laser heating^13^,^16^,^18^, in combination with a micro-engineered cell assembly with sample encapsulated by single-crystal materials^19^. A most recent SMP LH DAC study on the melting of Mo^13^, had reported a single data point at a pressure of 45 GPa, indicating a low-slope melting curve similar to other previous experimental studies. The study^13^ had relied only on the scanning electron microscope (SEM) observation of topological features on a quenched sample as the melting detection criterion; therefore, the result requires further scrutiny. Given the use of the recently developed SMP heating technique, the key remaining issue, preventing an accurate high-pressure Mo melting study, is the reliable identification of melting.

Melt detection using synchrotron X-ray diffraction, which is generally considered more reliable, usually depends on the *in situ* observation of a diffuse scattering-halo, indicative of the presence of liquid^20^. Unfortunately, the diffusely scattered X-ray signal is weak due to the miniscule volume of the liquid material produced in a LH DAC; its detection requires long X-ray collection times. State of the art studies on high *P–T* melting of other metals, which used fast X-ray diffraction^21^,^22^,^23^,^24^, still require X-ray collection time two orders of magnitude longer than the pulse duration of a typical SMP laser heating experiment. Due to this limitation, diffuse X-ray scattering has not been used with short pulse heating for melting detection.

The grain-size and its distribution in materials depend on many factors including pressure-temperature paths and heating or cooling rates. Using a LH DAC in combination with X-ray diffraction^25^ or *ex situ* SEM analysis^26^, several crystalline materials were shown to undergo a grain-size reduction, or nano-crystallization, when heated above the melting temperature and then cooled rapidly by abruptly switching off the laser power^25^,^26^. Conversely, annealing in a LH DAC (that is, high-temperature heating but below the melting point) was shown to increase the average grain size in polycrystalline materials^25^. The nucleation process from liquid metals is typically fast relative to the quenching rate^27^. However, the rate of grain growth could be comparable or even slower than the quenching rate in the experiment, thus leaving a signature of fine-grained microstructure as the evidence of the molten history (Fig. 1a). Therefore, analysis of microstructure can be used as a criterion of signifying melting of a material, provided that the quenching rate in the experiment is between material's nucleation rate and the grain growth rate. Experimentally, there are two clear advantages of this approach. One is its detection of positive signals; the fine-grained microstructure quenched from molten region is clearly visible in the X-ray diffraction patterns. The other is high sensitivity. Since the detection is from quenched samples, relatively long X-ray collection times can be applied for sufficient data statistics, even with short heating durations.

Here we show that with coarse-grained starting samples of Mo, when the laser pulse power is adjusted to above melting temperatures followed by fast quenching, continuous Debye diffraction rings are observed arising from newly crystallized fine grains of the sample (Fig. 2). We report the results of systematic measurements of the melting points (*T*_M_) of Mo at high pressures up to 130 GPa using this approach. Mo, a body-centred cubic (BCC) structured metal, is expected to have a simple *P–T* phase diagram—a single BCC phase up to pressures of nearly 700 GPa (refs 2, 6) and up to melting. However, a detailed analysis of the X-ray diffraction images reveals an unexpected new kind of microstructure transition in Mo at high pressure and at temperatures of approximately 400–500° below melting, yielding highly textured fine grains of BCC Mo. Several previous studies based on the structure zone model (SZM) have shown^28^,^29^,^30^ that a similar transition occurs in vapour deposited Mo thin films under near vacuum conditions. Here we demonstrate, for the first time, the applicability of the SZM mechanism under high *P–T* conditions. We discuss how this microstructure transition, together with several other experimental factors, is related to the previously reported anomalously low temperature melting of Mo in a LH DAC^10^,^11^,^12^,^13^. In addition, one melting point is reported for pure iron (Fe) at a pressure of 36 GPa, as a demonstration of the general applicability of the method (see the demonstration of the proposed methodology in Supplementary Methods).

## Results

### Complexity in Mo microstructures revealed by X-ray diffraction

**Figure 1:**
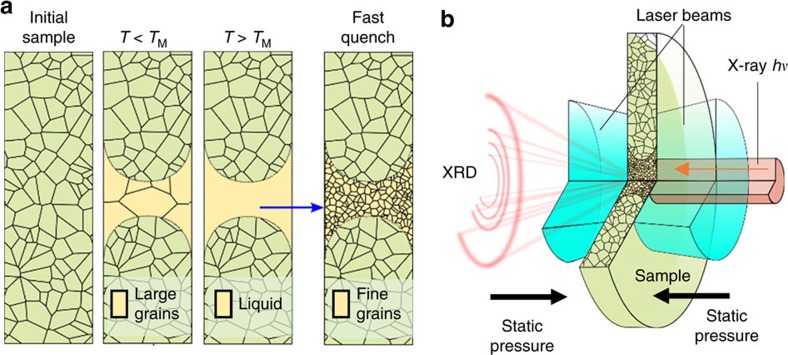
Microstructure signifying melting. (**a**) Cross-sectional schematic showing the Mo sample. Grain growth is promoted in the central region when temperature is below the melting temperature (*T*_M_); fine-grained microstructure emerges from the quenched molten region. (**b**) Cut-out view of the experimental design; the sample is irradiated by laser beams, concentric with the X-ray probe; X-ray diffraction from the heated volume of the sample is observed; external pressure-generating mechanism is not shown.

The LH DAC system with *in situ* microfocused X-ray diffraction at the High Pressure Collaborative Access Team (HPCAT)^16^, Sector 16 of the Advanced Photon Source, was used to generate the high *P–T* conditions and characterize the microstructure of *T*-quenched samples (Fig. 1b). X-ray diffraction images of the samples were recorded using a Pilatus 1M-F detector^16^. A typical experiment began by compressing a Mo sample to a desired high pressure. The high-temperature states at a given pressure were achieved, and X-ray diffraction images recorded iteratively; that is, the laser power of each subsequent heating pulse was adjusted incrementally, followed by an X-ray diffraction image recording for characterizing the quenched microstructure after each heating pulse. Due to the short heating duration of each pulse (5–20 ms), the changes in microstructure of Mo occurred in small increments, which allowed us to trace the evolution of discrete features in the X-ray diffraction images with temperature, as well as with time. At the various pressure points surveyed in this study, the main observations constitute histories of the quenched microstructures with temperature in the ranges between approximately 1,800 and 6,000 K, with intervals of approximately 10–50 K.

Initially, we expected to observe the grain coarsening where *T*<*T*_M_, and the appearance of fine-grained microstructure in samples where *T*>*T*_M_. In other words, we had expected to map out a melting line for Mo by grouping the X-ray diffraction images into two distinct kinds—showing grain coarsening before melting versus grain size reduction after melting. However, we found that the quenched microstructures consist of three distinct kinds, revealing a previously unreported microstructure transition in solid Mo at high *T* (referred to, here, as *T*_R_) below melting. In our LH DAC experiments, single-crystal magnesium oxide (MgO) was used as the thermal insulation layers adjacent to the Mo samples^19^. We found that the BCC Mo underwent an abrupt iso-structural change in crystallite size and orientation, from randomly oriented coarse grains to fine grains with biaxial orientation of BCC Mo with respect to the MgO. For clarity in presentation, the results of the microstructure transition and the melting of Mo are presented separately, below.

### Microstructural transition in molybdenum at high *P*–*T*

X-ray diffraction images of Mo samples quenched from incrementally increasing values of *T* (but keeping well below the melting point) showed incremental changes of the initial sample microstructure, displaying the growth of grain sizes (Fig. 3a). When Mo samples were subsequently heated to temperatures higher than *T*_R_, the X-ray diffraction images of these quenched samples showed an appearance of new preferentially oriented fine-grained features and a weakening of diffraction intensity from small *d*-spacing reflections. The X-ray diffraction images of the new microstructure showed in-plane angular alignment between the BCC Mo [(110), (200), (211)] and the face centred cubic (FCC) MgO [(200), (220), (311)] Bragg reflections, respectively (Fig. 3b), signifying a biaxially aligned texture. That is, Mo samples, quenched from the temperatures above *T*_R_ and below melting, contained new fine grains, preferentially oriented with respect to the MgO. The observation of the in-plane aligned texture demonstrates a near epitaxial crystallite growth mechanism in Mo at *T*>*T*_R_ (Supplementary Discussion). Successive heating of Mo by a sequence of heating pulses (at the same given temperature*, T>T*_R_) was seen to incrementally increase the amount of texture and reduce the X-ray diffraction from the randomly oriented coarse grains, and coarsening of the preferred oriented grains. In contrast, subsequent heating of the textured samples to *T<T*_R_ showed the return of a randomly oriented coarse microstructure, demonstrating the reversibility of the transition. We reason that this microstructural transition can be explained by the fact that large Mo grains tend to become unstable at high temperature, due to high atom mobility^28^,^29^,^30^, and reorganize into smaller crystal grains, according to the SZM^28^,^29^,^30^. We note, however, a more rigorous theoretical treatment is still needed to explain quantitatively the exact atomic processes. The presence of the oriented MgO crystals, which have a favourably matched lattice to that of Mo, enabled the newly formed Mo grains in the high-*T* structure zone to grow in a nearly epitaxial way, thus allowing the discrete structure zone boundary to be observable. In contrast, when single-crystal Al_2_O_3_ layers were used in several experimental runs, in place of MgO, the X-ray diffraction did not show any in-plane alignment between Mo and Al_2_O_3_ at *T*>*T*_R_. However, some weakening of the Mo signal from small *d*-spacing reflections was seen, indicating a presence of some degree of texture in the quenched microstructure. The experimental *P–T* conditions, which led to the observation of random versus textured microstructure in quenched samples, as well as the estimated microstructure transition boundary *T*_R_(*P*), are shown in Fig. 4.

We note that the observation of the microstructural transition was only possible when we used the single-crystal encapsulation layer. The lattice similarities in MgO and Mo at a specific orientation enabled the preferred orientation in Mo during re-crystallization process. Without the use of a single-crystal contact material, we would expect Mo grains to re-crystallize with random orientations after crossing the re-crystallization zone boundary.

A recent ‘flash' LH DAC study^13^ reported one Mo melting data point at 45 GPa and a temperature of 3,107 K using SEM observation of the topological surface features in the quenched samples. The samples in the ‘flash' study^13^ were heated while surrounded by a fluid pressure medium. We note that the *P*–*T* values for melting point^13^ coincide closely with the *T*_R_ values in this study (Fig. 4). It is possible that the abrupt changes in crystalline microstructure in Mo, that is, during the microstructure transition described above, can modify the topology of surface features on the samples if they are exposed to a fluid surrounding. Therefore, it is likely that the surface features, previously interpreted as melting^13^, were formed due to the microstructure change in solid Mo.

A hypothesis involving a shear-induced anisotropic plastic flow at high *P–T* has been proposed^31^ to account for the anomalously low melting temperatures observed in tantalum (Ta). Given that both Mo and Ta are of the same BCC structure, it is interesting to consider whether the plastic flow hypothesis could be used for the microstructure transition in Mo. The study in ref. 31 stipulates that the (110) peak should disappear in X-ray diffraction after the BCC Ta transforms into an anisotropic plastic flow under shear at high *P*–*T*, owing to the particular mechanics of the predominant BCC slip system. However, our *in situ* X-ray diffraction observations on Mo have shown that the Mo (110) peak does not disappear after the onset of the microstructure transition. Therefore, shear-induced plastic flow is unlikely an explanation for the microstructure transition in Mo at the experimental conditions of this study.

Furthermore, we propose that similar phenomena in several other controversial metals (Fe, Pb and Ta), termed by others as ‘fast re-crystallization'^22^,^23^,^24^, and sometimes attributed to grain rotation^22^,^23^,^24^, shear-induced anisotropic plastic flow (for Ta)^31^, or melting^32^, may instead be explained by the SZM. This hypothesis may be confirmed experimentally in specially designed LH DAC experiments with X-ray diffraction or grain orientation sensitive SEM analysis, provided that an appropriate preferred oriented and lattice-matched substrate can be implemented to facilitate the observation.

### High-slope melting curve in molybdenum

**Figure 2:**
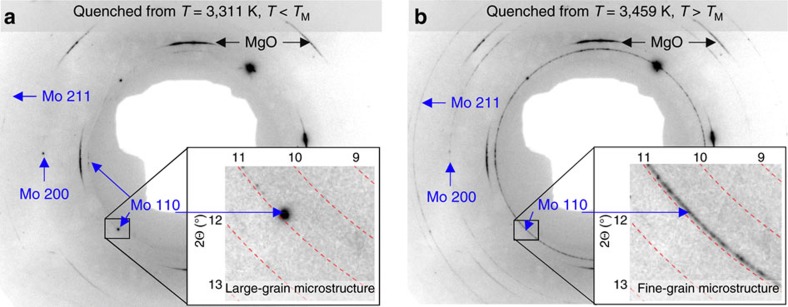
X-ray diffraction textures of samples quenched from below and above *T*_M_. (**a**) X-ray diffraction image collected after heating to a temperature of 3311(75) K, *T*<*T*_M_; and the presence of large Mo diffraction spots and the absence of continuous Debye rings signifying a large-grained microstructure. (**b**) X-ray diffraction image collected after heating to a temperature of 3439(75) K, *T*>*T*_M_; and the appearance of the continuous Debye rings of the new BCC Mo [(110), (200), and (211)] and disappearance of some Mo diffraction spots signifying melting during the heating pulse. [Pressure=31(3) GPa; X-ray *λ*=0.4066(1) Å].

**Figure 3:**
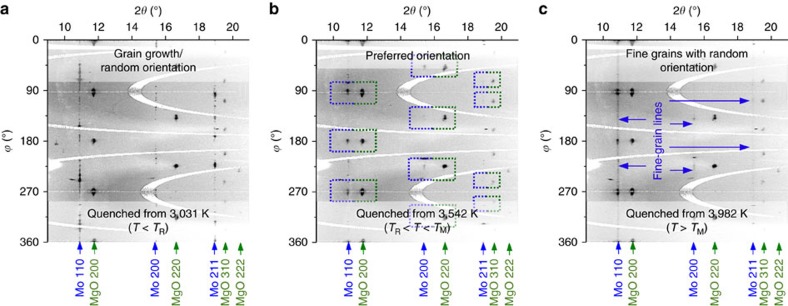
Azimuthally unwrapped X-ray diffraction images of quenched molybdenum. (**a**) Quenched from *T* below the microstructure transition, showing grainy, discontinuous Mo diffraction features, signifying grain growth; (**b**) Quenched from *T* above the microstructure transition and below melting, Mo diffraction features are preferentially oriented in *ϕ* angle in relation to diffraction spots from MgO; (**c**) Quenched from *T* above melting, continuous Mo diffraction features, showing fine-grained, randomly oriented microstructure signifying melting. (Pressure=43(4) GPa, X-ray *λ*=0.4066(1) Å).

After heating Mo to a sufficiently high temperature (several hundred degrees above *T*_R_) and quenching rapidly, fine-grained and continuous Debye rings were observed in the X-ray diffraction images, indicating randomly oriented grains. The appearance of fine-grained Debye rings was accompanied by an abrupt weakening or disappearance of Bragg reflection spots from one or several larger grains (Figs 2b and 3c; Supplementary Fig. 1). In some cases, not fully discontinuous Debye rings with random texture were recorded, likely due to the low volume of the quenched melt present in the sample (Supplementary Discussion). Concurrently, X-ray diffraction signal from small *d*-spacing reflections, which was suppressed in the textured samples at *T*>*T*_R_, was seen to re-appear abruptly, indicating a loss of axial preferred orientation. We emphasize that the observation of the contrasting features in X-ray diffraction images of the quenched textured microstructure versus solidified melted microstructure was facilitated specifically by the use of single-crystal MgO (as discussed in the previous section). It is possible that not every insulating material is appropriate for carrying out an X-ray diffraction study on the melting of Mo at high *P–T*.

One remaining question is whether the microstructure signature may arise from a solid–solid phase transition. We ensured the absence of a solid–solid phase transition from the *in situ* LH X-ray diffraction data. While detecting melting using the *in situ* LH X-ray diffraction diffuse scattering signal is challenging, obtaining evidence of a solid–solid phase transition using the *in situ* LH X-ray diffraction is feasible. In this study, *in situ* LH X-ray diffraction was used at temperatures corresponding to the observed changes in the quenched microstructure. The *in situ* LH X-ray diffraction data, collected concurrently with the heating pulse intervals (see Methods), did not show a solid–solid phase transition (a phase other than BCC) at any of the *P–T* conditions examined in this study.

**Figure 4:**
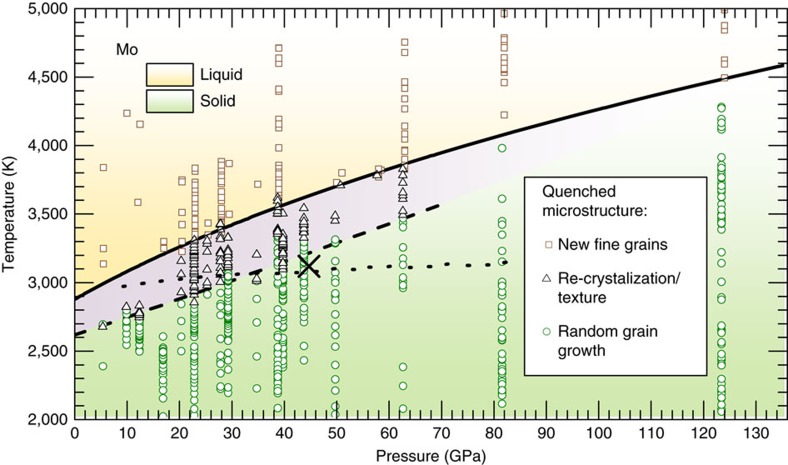
Experimental *P–T* conditions in this study. Grain growth (circles, green), re-crystallization with preferred orientation, that is, texture (triangles, black), new fine grains in microstructure, signifying melting (squares, brown). Dashed line—Mo microstructure transition (this work). Dotted line—Mo melting determined by previous LH-DAC studies (based on laser speckle method^10^,^11^ and X-ray diffraction^12^). Denoted by X is the optical observation of the quenched surface after ‘flash' heating^13^, is shown to coincide closely with the boundary of the microstructure transition as determined in our study. (Cold pressure values, instead of thermal-pressure corrected values, are shown for clarity (see ‘Thermal pressure' in Methods)].

The *P–T* conditions, which produced this fine-grained microstructure in the quenched samples, are presented in Fig. 4. The lowest *T* values, at any given pressure, which yielded the first appearance of a fine-grained and randomly oriented microstructure, were recorded as the melting temperature, *T*_M_(*P*). A total of 17 melting points *T*_M_ (*P*) at pressures up to 130 GPa, were used for fitting the melting curve using the Simon function,


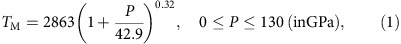


**Figure 5:**
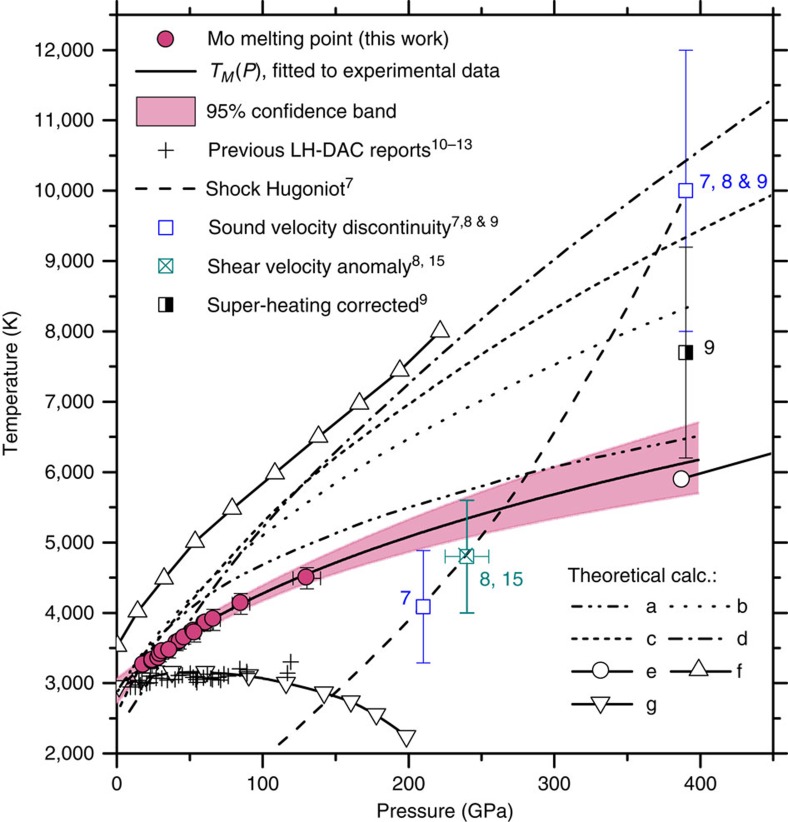
The melting curve of molybdenum under compression. Red filled circles—melting points obtained in this work by using the quenched microstructure criteria. The pressure error bars represent the range between the cold pressure and the EOS corrected pressure (see ‘Thermal pressure' in Methods); the temperature error bar estimates the spectroradiometry measurement uncertainty^16^. Previous LH DAC results^10^,^11^,^12^,^13^ show an essentially flat melting curve. Open squares—sound velocity discontinuities from shockwave studies^7^,^8^ measured along the calculated Hugoniot^7^,^8^,^9^ (long dash). Crossed square—shear bulk modulus anomaly^8^,^15^. Theoretically calculated melting curves and phase transitions: a—Belonoshko *et al*.^3^; b—Cazorla, *et al*.^4^; c—calc. melting from bcc phase, Belonoshko *et al*.^2^, d—calc. melting from fcc phase, Belonoshko *et al*.^2^; e—calc. BCC-FCC boundary, Belonoshko *et al*.^2^ and Zeng *et al*.^6^; f—Moriarty^5^; g—*d*-band-mediated melting, Ross *et al*.^14^

The fitted melting curve function was used to extrapolate the melting temperatures to 400 GPa; the result is shown in Fig. 5, overlaid with 95% confidence bands and other data from literature.

### Model system and accuracy of melting detection

**Figure 6:**
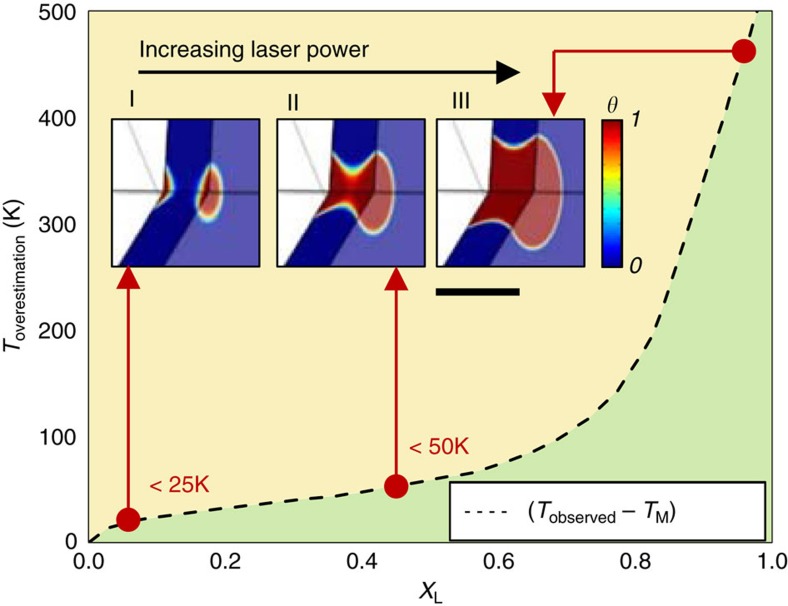
Liquid volume distributions at the onset of melting. (Stages I–III) The progression of the liquid volume distribution at increased heating laser powers. Dashed line: variation of *T*_overestimation_ (that is, *T*_observed_—*T*_M_) versus the parameter *X*_L_ (0≤*X*_L_≤1) (that is, the fraction of the diffracted signal intensity originating from the material quenched from a liquid state). Scale bar, 10 μm.

The key aspect of any method for studying high pressure melting is the reliable detection of liquid. Therefore, it is important to consider the limit of the liquid detection in our experiments and to quantify its effect on the resulting melting point determination. Here the detection limit is defined as the finite lower limit of the liquid volume present in the hot sample before its signature can be detectable in the quenched sample. In a LH DAC experiment with a fixed laser power distribution profile (near Gaussian function), the liquid volume can be made sufficiently large for positive detection by increasing the heating laser power (see stages I→II→III in Fig. 6). However, the high laser power increases the temperature of the liquid volume beyond the melting point, resulting in the recorded temperature higher than the true melting point. Therefore, the recorded temperature of the liquid with the minimum detectable volume is higher than the true melting point. Highly sensitive liquid detection will reduce the minimum detectable volume and consequently, the difference between the recorded temperature and the true melting point.

We have performed numerical heat flow calculations to quantify the systematic errors in melting point determination associated with liquid volume detection limitations (see the description of the numerical model in Supplementary Methods). Given that the X-ray diffraction is a bulk probe, that is, the diffracted signal originates from the total sample/X-ray beam cross-section (Fig. 1b), we define a dimensionless parameter *X*_L_ (0≤*X*_L_≤1), to refer to the fraction of the diffracted signal intensity originating from the material quenched from a liquid state. The observable parameter in the experimental system is the surface temperature of a circular area (4 μm in diameter) in the center of the hot spot^16^ (see Methods). Therefore, given the results of the heat flow model (see description of melt detection in Supplementary Methods), we have obtained the numerical relationships between the observed temperature *T*_obs_ and the true melting point temperature *T*_M_, given a range of detection limits, using the fraction parameter *X*_L_ (Fig. 6). This relationship can be used to assess quantitatively the temperature over-estimation associated with an X-ray diffraction based melt detection technique by making an appropriate estimation for minimal detectable *X*_L_ (Fig. 6). Because the X-ray diffraction images obtained in this study have a very high signal-to-noise-ratio due to the use of long X-ray collection times and a detector of high dynamic range and low readout noise, even weak signal (that is, continuous Debye rings, arcs), *X*_L_ as small as 0.05, is visually discernable with confidence. Given the low detection limit, the over-estimation in temperature should be less than ∼25 K (Fig. 6), which is comparable or less than the uncertainty level of spectroradiometry. However, we note that the consideration of the nucleation rate and the grain growth rate during the quenching were not included in the above numerical analysis due to the lack of such data for Mo at high *P–T* in available literature. The current treatment assumes that the effects of the nucleation rate and grain growth rate on the temperature over-estimation are small.

### Molar volume of molybdenum at high *P–T* below *T*_M_

Previous melting studies on other metals, Ta and Fe (refs 23, 24), have suggested that a plateau in molar volume as a function of temperature can be used as a melting criterion. However, in our study, we did not always observe the plateau upon melting of Mo. Due to the rapid grain growth, only a limited number of large grains could be observed, leading to poor statistics in *in situ* determination of the molar volume at high *T*. It is important to note that large crystals in the hottest region of the sample often were not observed by X-ray diffraction due to possible unfavourable Bragg conditions (Supplementary Fig. 2a), even though they may have been present (Supplementary Fig. 2b). In fact, less than half of the experiments showed a plateau or drop in the lattice parameter at temperatures near the observed melting. Although the lattice parameter plateau could sometimes be correlated with melting, we find that it is not a reliable melting criterion by itself and relying on volume data alone has the potential to cause large uncertainties in the *T*_M_.

### Pressure uncertainty

The actual pressure condition during the heating pulse in a DAC, just prior to melting (at *T*<*T*_M_), may differ substantially from the value determined before/after the heating pulse due to the thermal-pressure effects^33^. A large uncertainty in the pressure determination (that is, thermal pressure) at high *T* conditions, consequently, may lead to a significant uncertainty in the reported slope of the melting curve *T*_M_(*P*), and should be evaluated and constrained quantitatively. A calculation of the thermal pressure (see Methods) based on the Mo lattice parameters showed that the pressure during a heating pulse is 3–7% higher than the corresponding cold pressure, which is typical of the expected values in a LH DAC^33^. The melting curve of Mo reported here was obtained using the thermal-pressure corrected values.

### Molybdenum–carbon solid solution and its effect on *T*_M_

Long duration laser heating inside a DAC of materials with a strong carbon affinity is technically problematic due to the carbon diffusion from diamond anvils into the sample^34^. Mo is one of the materials well known to form carbides with a variety of stoichiometries and structural polymorphs^35^,^36^,^37^. Moreover, any form of Mo carbide is expected to melt at a lower temperature than pure Mo; therefore, it is important to consider a possible presence of carbide in the sample when studying the melting of pure Mo. We note that in several experimental runs (see description on Mo carbide in Supplementary Discussion), X-ray diffraction showed varyingly small amounts of an unexpected phase (Supplementary Fig. 3a) having a FCC crystal symmetry, which we attributed to a Mo carbide^35^,^36^,^37^ impurity. In contrast to the diffuse scattering method, the quenched microstructure criterion of this study enables the melting signature to be traced back to the appropriate phase by examining the corresponding Debye rings (that is, BCC Mo versus Mo carbide). By applying our quenched microstructure melting criterion to the carbide phase, we found that it had a nearly flat melting curve with *T*_M_=∼3,000 K, with a weak dependence on pressure. However, we note that the Mo carbide melting results presented here are preliminary, and a full study of the Mo carbide melting curve at high *P–T* is not in the scope of the current communication. We also found that in samples containing the carbide phase, melting of the BCC Mo phase occurred at temperatures approximately 100–150 K lower compared to samples which did not show any carbide contamination (Supplementary Fig. 3b) at similar pressures.

Pertaining to clarification of the discrepancy between our results and a previous LH DAC study of the Mo melting^12^ based on diffuse X-ray scattering, we note that the previous study^12^ used relatively long sample heating durations (several minutes at each pressure point), likely leading to strong carbon contamination. In fact, the X-ray diffraction patterns in the report^12^ do display features that are difficult to rule out as the presence of the carbon contamination. Therefore, it is possible that the diffuse scattering^12^ had originated from the liquid or amorphous Mo carbide, due to its low melting point at *T*=∼3,000 K. Similarly, the preceding studies^10^,^11^ on melting of Mo, which relied on the visual observation of ‘speckle motion', and/or temperature versus laser power plateaus as the melting criteria, also used prolonged heating of Mo at high *T*; therefore, their results, in addition to being affected by the microstructure transition, may also have been affected by the Mo carbide contamination.

It should be noted that most of our samples did not present a distinct carbide phase because of the short heating duration. However, on comparing the equation of state of (EOS) of Mo and MgO in quenched samples, we saw that the samples quenched from higher temperatures showed a slightly higher volume than expected. Therefore, we cannot completely rule out possible carbon contamination as a solid solution in the BCC Mo lattice. Given that the contamination lowers the melting point of the coexisting Mo phase, our results should then be the lower bound of the melting curve of pure Mo. That is, the true Mo melting curve may be higher than what is reported here.

## Discussion

Our result for the melting curve of Mo shows a high slope, in drastic contrast to the earlier LH DAC studies^10^,^11^,^12^,^13^, which all produced an essentially flat functional behaviour of *T*_M_ with respect to pressure. Consequently, the extrapolation of our melting curve to higher pressures of up to 400 GPa, in contrast to earlier LH DAC results, is in far better agreement with reports on Mo under shockwave compression^8^,^9^,^15^,^38^. However, our result and the newer shockwave data^8^,^9^,^15^,^38^ both point to a lower melting temperatures than those predicted by *ab initio* molecular dynamics (MD) calculations^2^,^3^,^4^,^5^,^6^ (Fig. 5).

Earlier LH DAC studies^10^,^11^,^12^ all produced a flat slope melting curve with *T*_M_ between 2,900 and 3,200 K up to the pressure of 119 GPa (Fig. 5). As we have shown in the preceding sections, several phenomena, including the microstructure transition and carbon contamination, are possible explanations for the anomalously low melting temperature observed in earlier LH DAC studies^10^,^11^,^12^,^13^. It then follows that our results do not support the calculation results using a semi-empirical model of *d-*electron band mediated melting^14^, which had predicted a maximum in the melting curve at a pressure of 40 GPa—followed by a decrease in the melting temperature at higher pressures. However, it would be interesting to understand whether *d*-electron band contribution is needed to explain the instability of large Mo grains above *T*_R_, that is, the microstructure transition.

Regarding the melting points based on the sound velocity discontinuities in shockwave Hugoniot experiments, we note that the origins of the observed discontinuity points along the shock Hugoniot of Mo are still actively debated in the literature, with several publications and comments published in recent 2 years^8^,^9^,^15^,^38^,^39^. Hixson *et al*.^7^ first reported two sound velocity discontinuities on the Hugoniot, with the lower pressure discontinuity at *P*=210 GPa and *T*=4,090±800 K, assigned to a hypothesized solid–solid transition^7^, and the higher pressure discontinuity at *P*=390 GPa and *T*=10,000±2,000 K, assigned to melting^7^,^9^. However, a number of more recent shockwave experiments failed to detect the first discontinuity^7^ at the lower pressure, suggesting that it could be an artifact in the earlier experiments^8^,^9^. The most recent shockwave-and-X-ray diffraction combined study (SW X-ray diffraction)^9^, based on the observation of the Mo X-ray diffraction (110) line broadening, has suggested^9^ that the melting point should be 7,700±1,500 K, at *P*∼390 GPa, if a correction for the superheating of Mo prior to melting is applied. This result agrees with our extrapolated melting point within the experimental and extrapolation uncertainties (Fig. 5).

Interestingly, a recently published comment^15^ to a reported shockwave sound velocity measurement on Mo^8^ had proposed a re-analysis of the reported sound velocity data^8^, based on an analytical model^40^. It has been argued that, according to the data provided^8^, the melting should be interpreted to occur at the onset of the shear modulus drop, instead of the point of a sharp sound velocity discontinuity. Such a re-analysis^15^ of the sound velocity data^8^, based on the shear modulus anomaly, produced a melting point of 4,850 K, with an uncertainty of ±800 K at 240±15 GPa on the shock Hugoniot. We note that an extrapolated melting point of this study, at 240 GPa and 5,300±250 K, agrees remarkably well with the onset of the shear modulus softening of the shockwave data^8^,^15^,^38^ (Fig. 5).

The reported melting temperatures from theoretical *ab initio* MD simulations^2^,^3^,^4^,^5^,^6^ are all higher than our results (Fig. 5), with the exception of the study in reference^3^, which is close to our measurements. In fact, these MD results, except reference^3^, appear to be more consistent with the earlier higher *P–T* discontinuity point on the Hugoniot^7^,^8^,^9^. However, as discussed above, the most recent SW X-ray diffraction study^9^ has suggested a lower melting temperature—in better agreement with our result. The most recent MD study^39^ does point out that larger-scale MD calculations are needed to identify precisely the effects in Mo at the highest *P–T* conditions. Our results call for more theoretical studies to include the SZM re-crystallization at high *P–T* for a better understanding of the complex behaviour of Mo and other transition metals.

## Methods

### Sample preparation

Mo foil (99.95%) was purchased from Alfa Aesar, manually thinned and cut into pieces of required dimensions using steel and tungsten micro-tools. MgO (100 μm, <100> plane orientation) and Al_2_O_3_ (100 μm, <0001> c-plane orientation) single-crystal plates were obtained from MTI Corporation. Rhenium DAC gaskets, as well as MgO and Al_2_O_3_ parts for the sample encapsulation assembly, were prepared using the laser micromachining system at HPCAT^19^.

### Molybdenum sample assembly with single-crystal MgO encapsulation

**Figure 7:**
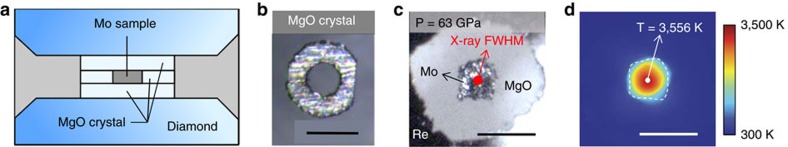
Typical sample assembly and geometry at high pressure and high temperature conditions. (**a**) Schematic drawing of sample encapsulation. The middle layer secondary gasket, laser-cut from a 15 μm thick sheet of single-crystal MgO (also shown in **b**), prevents the sample from spreading out radially during axial compression. (**b**) Microscope photograph of a laser-machined MgO crystal micro-gasket, typical thickness ≈ 10 μm. Scale bar, 100 μm. (**c**) Microscope photograph of the sample chamber as viewed through the diamond window. Red circle in the middle of the molybdenum sample denotes the FWHM of the X-ray probe profile (see Methods). Scale bar, 50 μm. (**d**) Image of temperature distribution collected during a typical heating at a pressure of 63 GPa; the temperatures are recorded the point of circular area (4 μm in diameter), denoted by white circle; the outline of the sample is denoted by the dashed curve. Scale bar, 50 μm.

The Mo foil was mechanically pressed and cut to produce plates of dimensions ranging 20–40 μm on the side and 3–10 μm in thickness. MgO single-crystal plates were polished to reduce in thickness to ∼15 μm using diamond lapping film. Thermal insulation layers (that is, discs and micro-gasket encapsulation assembly) were prepared by micro machining the polished MgO plates^19^ (Fig. 7a,b). In several of the experimental runs, Al_2_O_3_ disc was used as the insulation layers. Micro machined MgO insulation layers were heated in an air furnace above 1,000 °C in order to burn off impurities, particularly Mg(OH)_2_. Sample and insulation layers were stacked inside a cylindrical hole in a pre-indented rhenium gasket^19^. The entire DAC assembly was heated to above 100 °C, in a glovebox (Innovative Technology PL-2GB-IL-GP1) with <0.1 parts per million (p.p.m.) H_2_O, in order to remove any adsorbed water from the sample assembly, immediately prior to sealing the DAC pressure vessel (Fig. 7c). Symmetric type DAC in combinations with cubic-BN seats and diamonds with culet diameters between 300 μm and beveled 150–300 μm were used in this study to generate high pressure. Raman spectroscopy was performed, after experiment, on recovered samples to check for a possible formation of MgMoO_4_. No MgMoO_4_ Raman signal was detected, indicating that the MgO insulating layer remained chemically inert during the experiment. See additional notes including the re-use of samples in multiple runs in Supplementary Table 2 in Supplementary Methods.

### X-ray diffraction set-up

X-ray diffraction of the central volume of the Mo plate was performed in axial geometry using micro focused synchrotron X-rays, 4–6 μm vertical by 5–7 μm horizontal full width at half maximum (FWHM)^16^ (Fig. 7c). Pilatus 1M-F detector was used to collect diffraction images in the majority of the experimental runs, except for one experimental run wherein the Pilatus 1M detector was unavailable and a Mar345 CCD area X-ray detector was used instead. A range of 3 to 15 s of X-ray diffraction collection time were used for obtaining the area diffraction images of the quenched samples at ambient temperature conditions. Radial integration (binning) was performed on the area diffraction images using the Dioptas software^41^. *d*-Spacings of several Mo and MgO reflections were calculated by fitting Pseudo-Voight functions to corresponding peaks in the radially integrated diffraction patterns. Lattice volumes of Mo were calculated by averaging the *d*-spacing from each of the observed *hkl* lines.

### High temperature generation and spectroradiometry

The sample was irradiated from two sides simultaneously with slightly de-focused, co-axial, infrared laser beams. The shape of the incident laser-power distribution at the sample surface could be approximated by a Gaussian distribution. The FWHM of the laser power distribution was adjustable and in the range of 20–55 μm in diameter. The laser power was applied as single, square-modulated, manually triggered pulses, in the range of 5–20 ms in duration (Supplementary Fig. 5). A sequence of laser pulses was applied, each with incrementally larger laser power. A microscope system with a gated CMOS camera, as previously described^16^, was used to collect *in situ* 2-dimensional (2D) images of the thermal radiation from the laser-irradiated sample surfaces (Fig. 7d) during the heating pulse. The information in the 2D images was used for adjusting the heating laser to align the heating spot with the spectrometer pinhole for temperature measurement. The initially set lasers' focus position and size were adjusted, in order to obtain precise alignment of the center of the hot spot, the temperature measurement and the X-ray focus positions. The position of the sample with respect to the X-ray focus and laser positions were also adjusted, if necessary, in order to optimize the symmetry of the hot spots on both sides of the sample. After such initial adjustments, the position of the sample relative to the X-ray beam was kept constant throughout the remainder of the given experimental run; however, the focus size and position of the laser beams with the respect to the sample were continually adjusted if deemed necessary. Thermal radiation in the range of 600–800 nm was recorded from a circular areas of 4 μm in diameter (Fig. 7d, Supplementary Figs 6a and 7b), axially aligned with the X-ray focus on both sides of the sample, as described previously^16^. Thermal radiation collection was timed to coincide with the duration of the particular laser pulse (Supplementary Fig. 5). In cases when very high temperature was to be reached, the thermal radiation collection times were made shorter than the duration of the laser pulse, in order to avoid the saturation of the counts on the detector. In several of the experimental runs, thermal radiation was measured multiple times during a given heating pulse in order to obtain a time-resolved measurement (Supplementary Figs 5 and 6, also see discussion of sample temperature response in Supplementary Methods). Temperature was calculated by fitting the parameters of the Planck equation. Greybody radiation with wavelength independent emissivity was assumed.

### X-ray diffraction measurements from hot and quenched samples

After initial laser alignment, heating pulses with incrementally increasing values of laser power were applied the sample. X-ray diffraction images were recorded from the sample at high temperature, *in situ*, using a gated X-ray detector^16^. After each heating pulse, an X-ray diffraction image was collected from quenched sample volume after each heating pulse using a relatively long collection time (3–15 s). Lattice volumes of Mo at given high-temperature conditions, and correspondingly quenched to ambient temperature, were calculated using the approach described above. Logarithmic scaling was applied to the X-ray counts in the diffraction images in order to improve the visual contrast of the diffuse low-count features. X-ray counts in the diffraction images obtained from quenched samples were mapped to a *ϕ*-2*θ* geometry for improved visual observation of quenched texture phenomena in Mo such as grain growth, re-crystallization and melting.

See additional discussion on the validation of the microstructure method for melt detection in Supplementary Methods (Supplementary Figs 10 and 11).

### Thermal-pressure

As a first approximation, the thermal pressure was calculated by directly using the observed lattice parameters (see ‘X-ray diffraction set-up' section) in conjunction with a Mo equation of state (EOS) from literature^42^,^43^.

This approximation produced pressures during the heating pulses (at *T*<*T*_M_) with values 5–10% higher than the corresponding cold pressure. It should be noted, however, that the spatial distribution of the X-ray probe was FWHM=5–7 μm (width-at-10%-of-maximum=12–18 μm), which is comparable in size to the width-at-90%-maximum of the temperature distribution in the sample during the heating pulse. Therefore, the recorded X-ray diffraction originated from hot as well as some cold parts of the sample—leading to a substantial over-estimation of the thermal pressure. With this consideration, we estimated the actual pressure during the heating pulse to be between the cold pressure and the apparent EOS corrected pressure. The pressure uncertainty was defined to be the range between the cold pressure and the EOS corrected pressure.

### Data availability

The data that support the findings of this study are available from the corresponding authors upon request.

## Additional information

**How to cite this article:** Hrubiak, R. *et al*. Microstructures define melting of molybdenum at high pressures. *Nat. Commun.* **8,** 14562 doi: 10.1038/ncomms14562 (2017).

**Publisher's note:** Springer Nature remains neutral with regard to jurisdictional claims in published maps and institutional affiliations.

## Supplementary Material

Supplementary Information Supplementary Figures, Supplementary Tables, Supplementary Discussion, Supplementary Methods and Supplementary References
