# Supplementary material for: Microstructures define melting of molybdenum at high pressures
Source: Nat Commun. 2017 Mar 1;8:14562. doi: 10.1038/ncomms14562 (PMC5337970; doi:10.1038/ncomms14562)
Supplement: Supplementary Information — Supplementary Figures, Supplementary Tables, Supplementary Discussion, Supplementary Methods and Supplementary References [file ncomms14562-s1.pdf]

Supplementary Figures

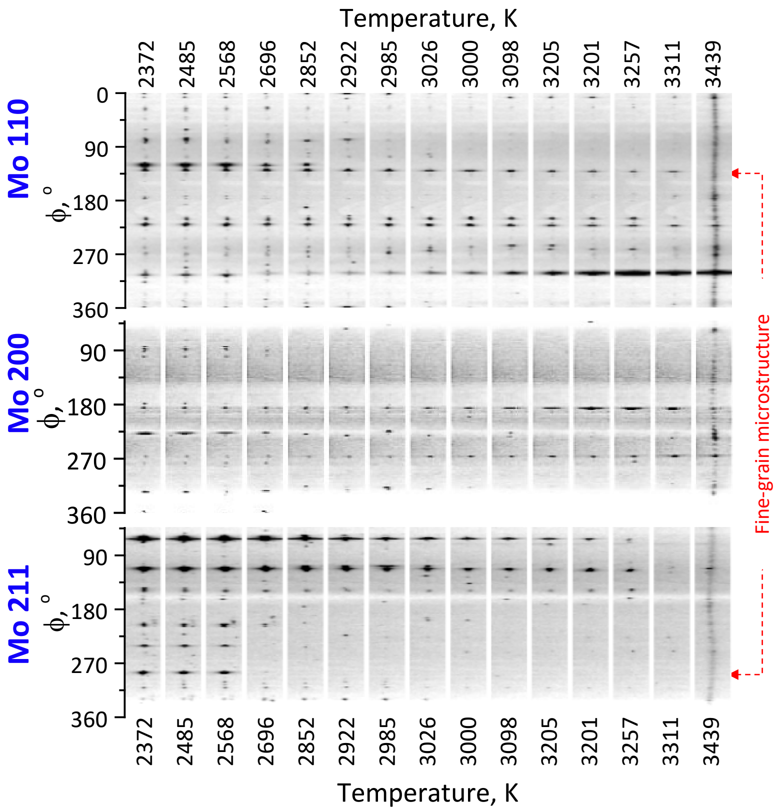

Supplementary Figure 1. Microstructure evolution seen in a sequence of azimuthally unwrapped XRD images of several Debye rings. The Debye rings, corresponding to molybdenum 110, 200, and 211 *hkl* reflections were recorded from temperature-quenched samples at a pressure of 40 GPa. Incremental grain coarsening is signified by decrease in number of spots up to the temperature of 3311 K; abrupt appearance of fine-grain microstructure is evident, on the last of the images by the observation of a continuous Debye line.

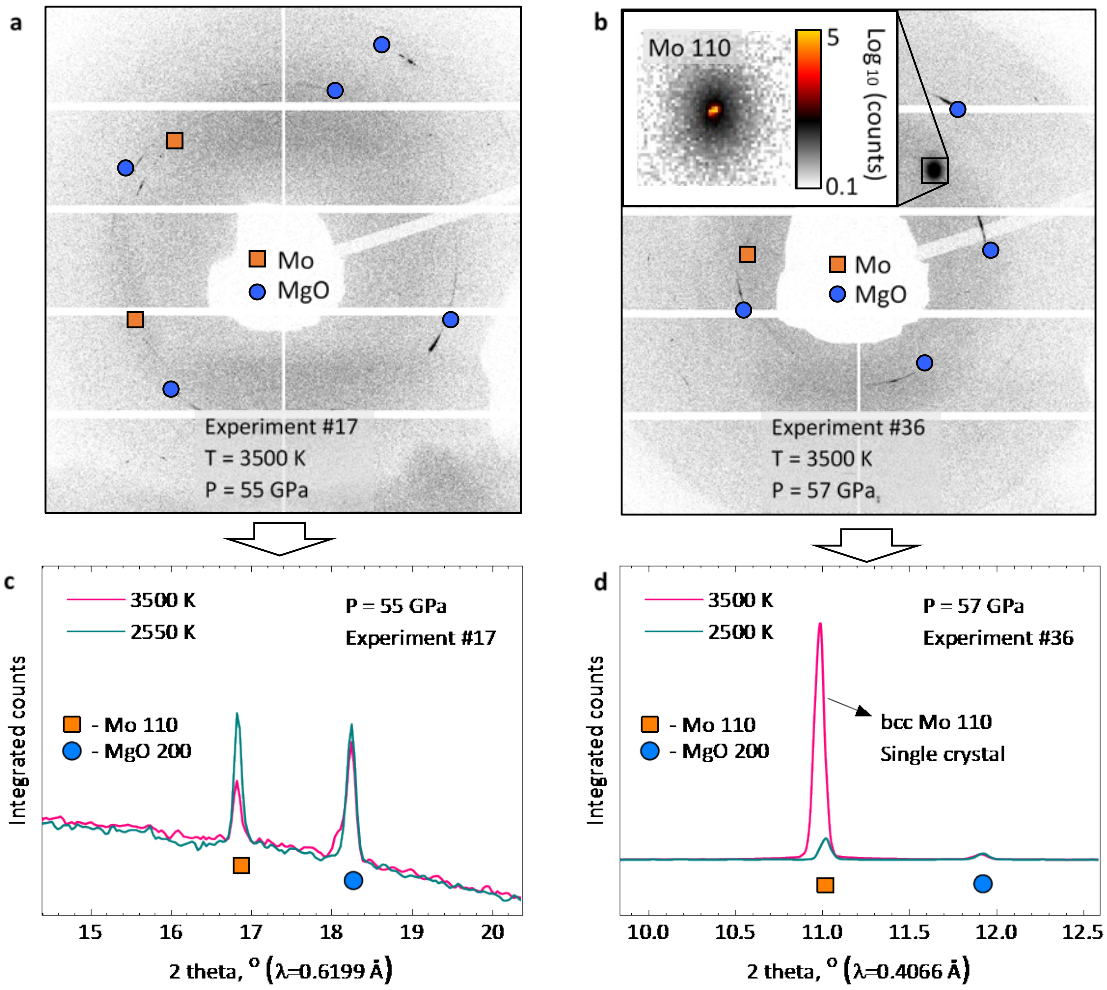

Supplementary Figure 2. Poor statistics of typical *in-situ* XRD due strong grain growth in Mo at high temperature. (**a**) In-situ XRD signal from a sample at 3500 K showing weak diffraction Mo and the surrounding MgO. (**b**) In-situ XRD signal from a different experiment, but at nearly identical conditions as (**a**), showing single Bragg reflection spot with extremely high photon count (insert). (**c**) Integrated counts vs. 2theta from (**a**) comparing the Mo diffraction at low and high temperature (2550 K and 3500 K), the Mo diffraction signal is seen weakened at high temperature. (**d**) In contrast to (**c**), the crystalline diffraction on Mo (integrated from **b**) appears significantly stronger at higher temperature (3500 K) compared to the lower temperature (2500 K).

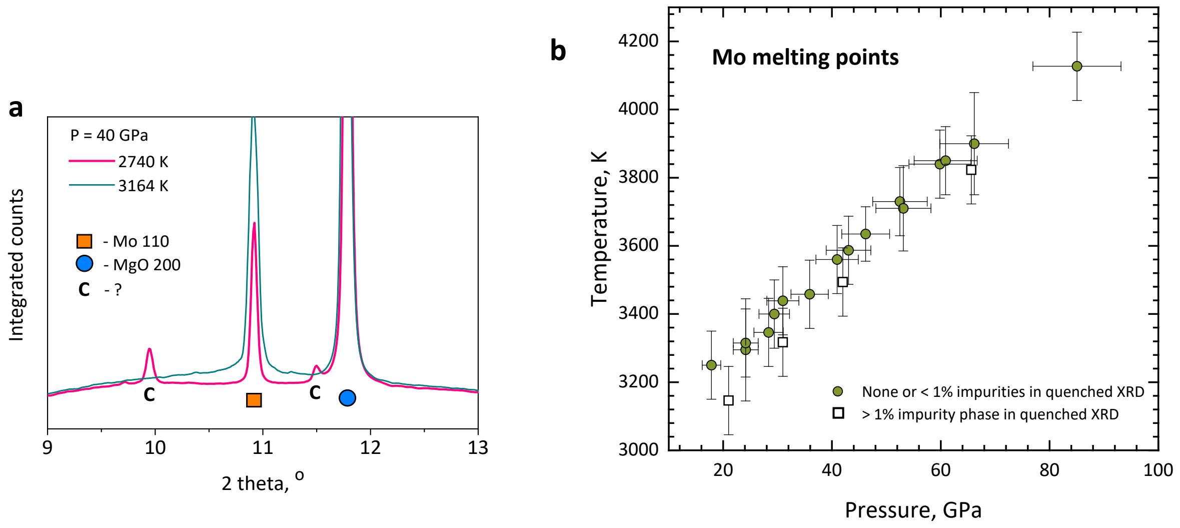

Supplementary Figure 3. Mo carbide phase and the consequent lowering of the Mo melting point. (**a**) XRD of a particular Mo sample quenched from a temperature of 2740 K, showing the formation of Mo carbide phase **C**; the same sample, consequently quenched from 3164 K shows the disappearance of crystalline carbide peaks, signifying the melting of phase at and above this temperature. (**b**) Systematic lowering of the melting points of the bcc Mo phase in the presence of Mo carbide phase. The percent of the phase, when seen, was estimated based on the relative intensity of bcc Mo and Mo carbide phase XRD peaks. [Pressure error bar: range between cold pressure and thermal EOS corrected pressure; temperature error bar: spectroradiometry measurement error].

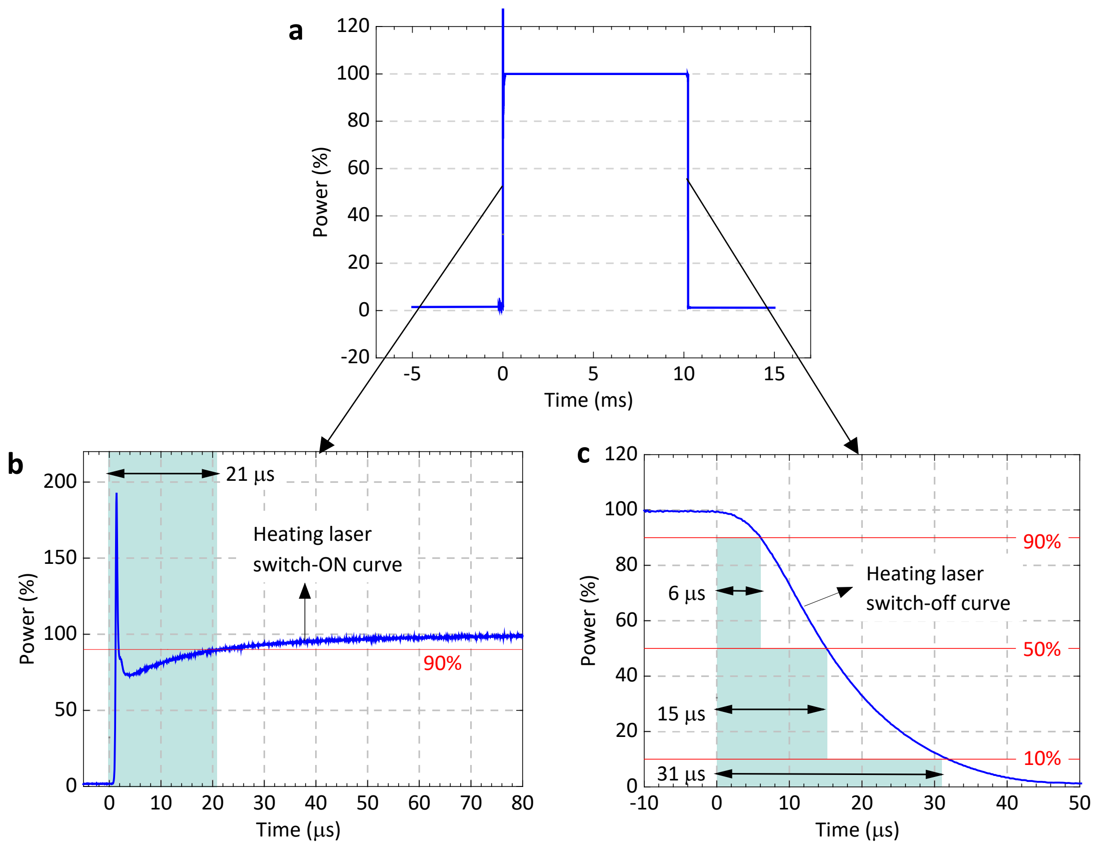

Supplementary Figure 4. Illustration of the IR heating laser modulated pulse shape. (a) Time dependence of the output power of the square modulated IR laser. **(b)** Switch-on characteristic of the IR laser. Note the switch-on power spike, i.e. the typical ~200% power overshoot. **(c)** Switch-off characteristic of the IR laser (90%, 50% and 10% power levels).

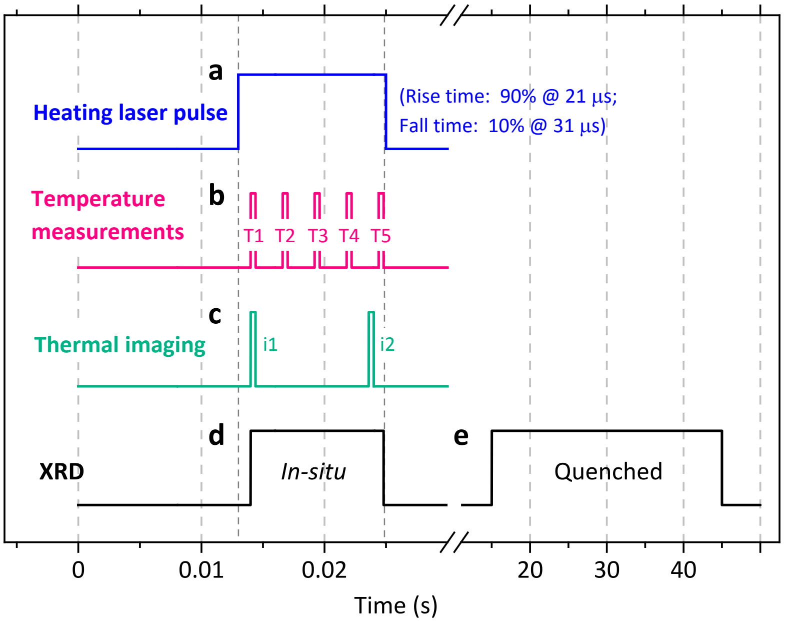

Supplementary Figure 5. Boxcar illustration of the pulse heating operation. Each laser heating pulse (**a**) was synchronized with one or several temperature measurements (**b**) (up to 20 per pulse), one or two thermal imaging measurements (**c**), and one in-situ XRD measurement (**d**). A long-exposure XRD (**e**) (10-30 seconds) was collected after each heating pulse to examine the quenched microstructure.

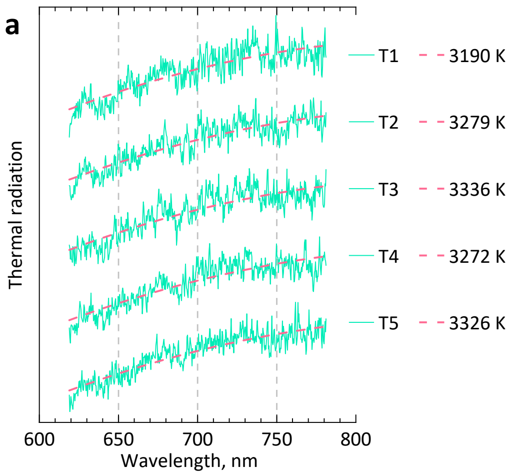

Supplementary Figure 6. Time resolved temperature measurements during a single heating pulse. (**a**) Offset plot of the thermal radiation signal collected at 5 discrete time intervals (Supplementary Figure **5b**) during a typical heating pulse, overlaid with Planck equation fits; (**b**) Temperature measurements at times T1-T2, error bars represent the standard deviation of the Planck equation fits.

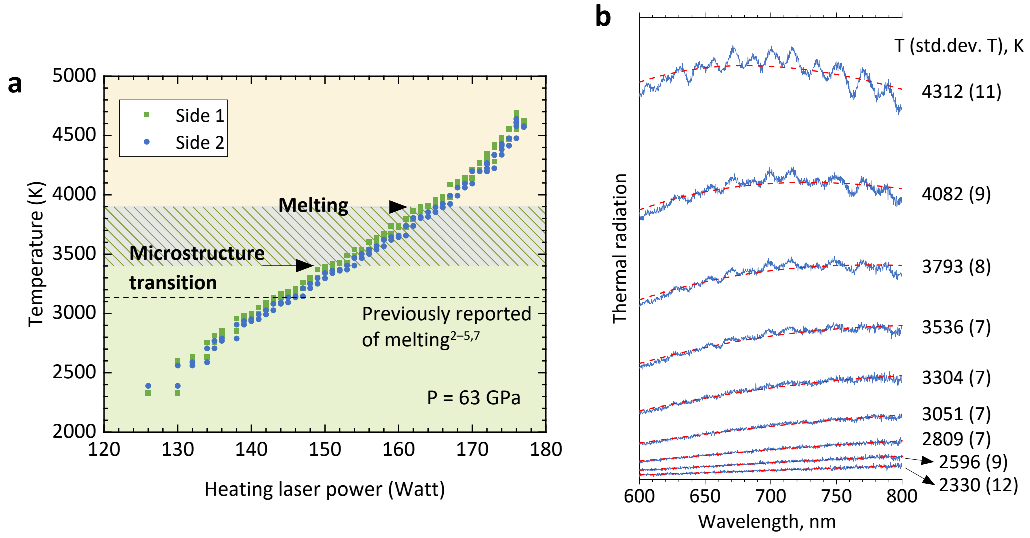

Supplementary Figure 7. Temperature conditions during discrete heating pulses in a typical experiment at a given high pressure. (**a**) Temperatures (sides 1 and 2 of the sample) vs. laser power plot shows a near linear dependence in molybdenum in LH DAC. Temperature of the previously reported melting point[2–5,7] at this given pressure is overlaid (dashed line). (**b**) Typical thermal radiation signal and Planck equation fits at several temperature points (absolute scale, arb. unit).

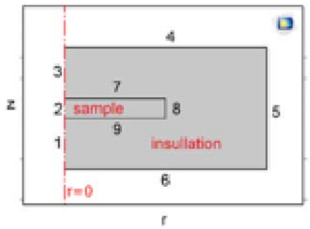

Supplementary Figure 8. Schematic of the geometrical model of the DAC setup used in numerical heat flow model. Radial symmetry around line *r*=0 is assumed. Numerical labels correspond to boundary conditions listed in Supplementary Table I.

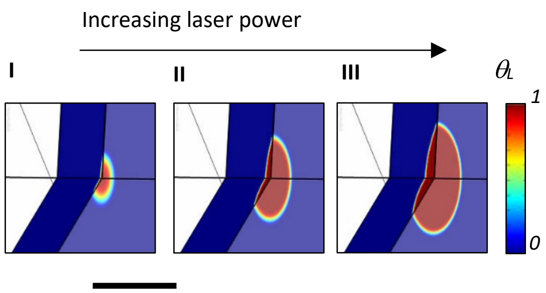

Supplementary Figure 9. Cutout schematics of liquid volume distributions for the case of single-side heated LH DAC. Stages **I-III** show the liquid volume distribution with increasing values of heating laser power. The liquid is localized only at the surface at which the laser power is incident, compare to Figure 6 I-III in the main text. (Scale bar = 10 μm)

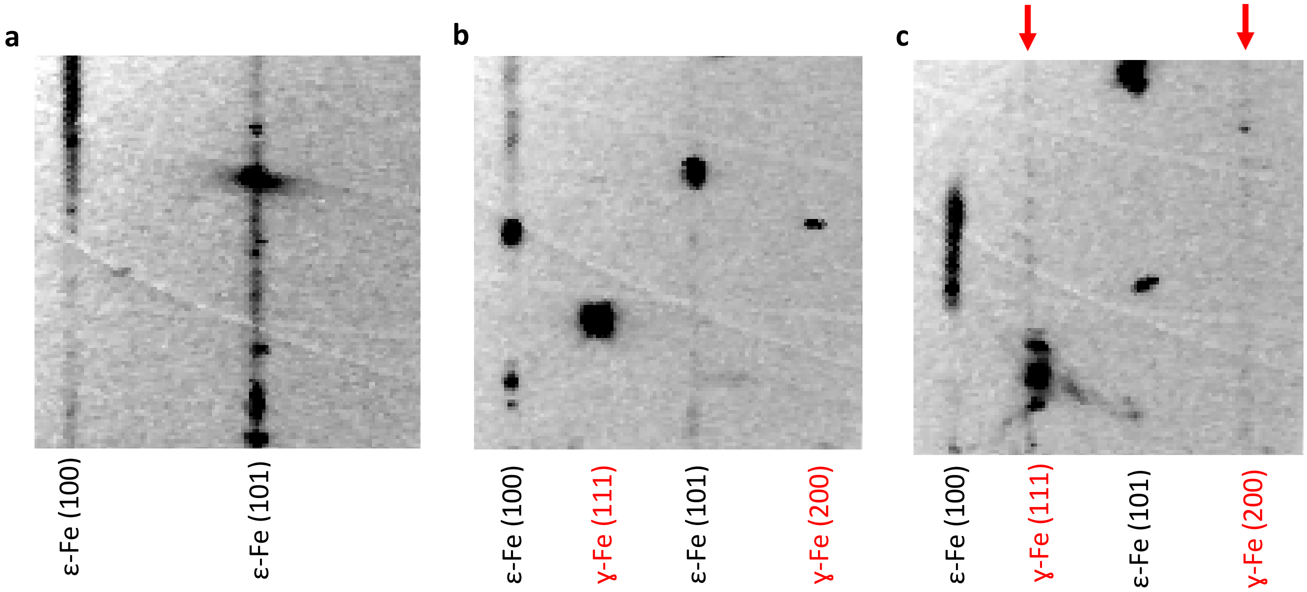

Supplementary Figure 10. Azimuthally unwrapped XRD textures of Fe samples quenched from $T<T_M$ and $T>T_M$. (**a**) XRD image collected after heating to a temperature less than 1610 (100) K, $T<T_{\varepsilon-\gamma}$, showing ε-Fe diffraction [HCP (110) and (101)], the ε-Fe [hcp (002)] is not observed due to preferred orientation. (**b**) XRD image collected after heating to a temperature less than 2550(100) K, $T<T_M$; the presence of large γ-Fe diffraction spots [HCP (111) and (200)] and the absence of continuous Debye rings signifying a large-grained microstructure. (**c**) XRD image collected after heating to a temperature of 2760(100) K, $T>T_M$; and the appearance of the continuous Debye rings corresponding to new γ-Fe [FCC (111) and (200)] fine-grained microstructure (annotated by red arrows) – signifying melting during the heating pulse. [Pressure = 36(3) GPa; X-ray λ=0.4066(1) Å]. MgO diffraction is not seen in the regions of interest (**a-c**) due the specific orientation of the MgO single crystal layers.

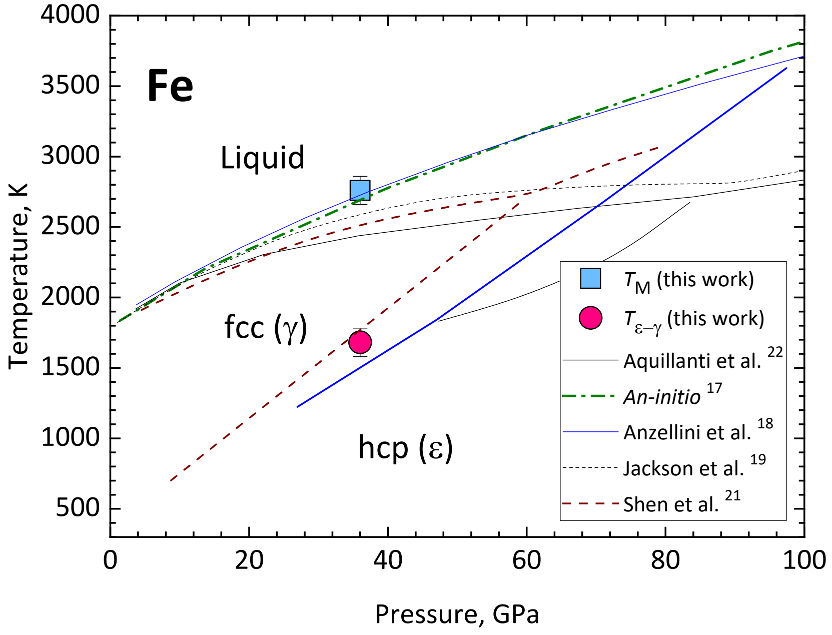

Supplementary Figure 11. High-pressure phase diagram of Fe showing T_M and $T_{\varepsilon-\gamma}$ obtained using the quenched microstructure approach (this work) overlaid over selected data from literature[17,18,20–22]. The temperature error bar represents the spectroradiometry measurement uncertainty[1].

Supplementary Tables

Supplementary Table 1. Boundary conditions used in solving the heat equation

No.	Boundary (refer to Supplementary Figure 8)	Dirichlet boundary condition
1	1, 2, and 3	$\boldsymbol{n} \cdot (k\nabla T) = 0$
2	4, 5, and 6	$T = T_0$
3	7, 9	$\boldsymbol{n} \cdot (k\nabla T) = \frac{Q_\mathrm{b}}{2\pi\sigma^2} \exp\left[-\frac{1}{2}\left(\frac{r}{\sigma}\right)^2\right]$

Q_b is the boundary heat source (laser power); σ is the width of the laser beam Gaussian distribution.

Supplementary Table 2. Sample notes

P (GPa)	T_M (K)	Sample note
17.9	3250	Fresh sample
24.2	3295	Fresh sample
24.2	3315	Fresh sample
28.4	3346	Fresh sample
29.4	3400	Second run on sample
31.0	3439	Second run on sample
41.0	3560	Fresh sample
43.1	3587	Second run on sample
46.2	3635	Fresh sample
52.5	3730	Third run on sample
59.9	3840	Fresh sample
60.9	3850	Fresh sample
66.2	3900	Fresh sample
85.1	4127	Fresh sample
35.9	3458	Fresh sample
53.1	3710	Fresh sample
130.2	4493	Fresh sample

Supplementary Discussion

Observations of the microstructure transition and melting

As we introduced in the main text, our method of liquid detection depends on the observation of re-crystallized fine-grained microstructure in the quenched sample. Therefore, in order for the new fine-grained microstructure to be observable, we found that sample material must first be made to undergo sufficient grain growth. That is, without sufficient grain growth, the XRD signal from new fine grains may be obscured by the signal from the original fine-grained microstructure of the sample. Grain growth in the samples was promoted by laser annealing at sufficiently high temperature, using either continuous-wave (CW) or SMP modes.

As we noted above, the successful observation of new fine-grained microstructure from quenched melt depends on sufficient prior re-crystallization of the sample. In subsequent analysis, we found that the samples at above 80 GPa were not re-crystallized sufficiently before melting; therefore, in order to clearly observe the new fine-grain microstructure, we performed an image background subtraction (using previous XRD image as a background for the XRD under examination). Therefore, the two data points at the highest pressure were arbitrarily assigned less weighed in the fitting of the melting curve function. We have also performed a separate fit of melting curve function with the highest pressure points excluded, and confirmed that such a fit did not deviate significantly from the fit obtained using all of the experimental points.

The lowest temperature showing the appearance of randomly oriented fine-grain microstructure was recorded as the melting temperature at a given pressure.

An example of a typical observed sequence of grain growth including the highly textured fine-grain microstructure transition at 300 to 400 K below melting, followed by an abrupt appearance of a new fine-grained microstructure after melting, is shown in Supplementary Figure 1.

Microstructure transition and preferred orientation in molybdenum

In some of the experimental runs, the two MgO crystals of the thermal insulation layers were closely aligned with respect to each other, and in some cases, they were not. The diffraction from MgO, at sufficiently high pressures, spread out over some range of the azimuthal angle, indicating the breaking of the single crystal layers into smaller crystallites oriented in the approximately the same orientation as the starting single crystal. In cases where the MgO crystals were closely aligned, the XRD patterns from each of the layers overlapped. In cases where the two starting MgO crystal layers were not aligned in the same orientation, we could distinguish the XRD patterns from each of the layers. In these cases, where the two MgO layers were misaligned with one another in azimuthal orientation, we then observed two distinct groups of Mo (110), (200) and (211) reflections – aligned with both groups of MgO reflections respectively, above the microstructure transition temperature. The fact that Mo aligned with both MgO layers indicates that the Mo alignment is a Mo-MgO boundary phenomenon, which can be explained by an epitaxial growth.

Molybdenum carbide

In a few cases, we found that the crystalline XRD from a minor Mo carbide phase emerged in samples quenched from T > ~2600 K. The XRD of this Mo carbide phase, in samples quenched from T > ~3000 K, showed no crystalline XRD peaks, however, presented as a single weak and diffuse Debye ring. This signified the melting of the phase. Further annealing of the sample at T < ~ 3000 K, the carbide phase with crystalline diffraction peaks re-appeared. The existence of the minor Mo carbide phase has the slight effect of lowering the Mo melting point as shown by the open squares in Supplementary Figure 3b.

Supplementary Methods

Laser system

The experiment described here used a laser-heated diamond anvil cell (LH DAC) setup with *in-situ* synchrotron x-ray micro-diffraction probe and double-sided heating, as described elsewhere[1]. The laser was electronically modulated to follow a square-shaped power profile (Supplementary Figure 4a). We noted that in the beginning of the square pulse output there is a power-overshoot, of 1-2 μs in duration, typical for the type of laser used in the LH DAC system (Supplementary Figure 4b). However, the power overshoot of the laser does not lead to a temperature overshoot in the sample due to the sufficiently short time scale comparing to the time scale for thermal response of the sample in the experimental setup used (See Sample temperature response section).

A typical experiment

In our experiment, the pulses of laser, temperature measurement, thermal imaging and in-situ XRD were synchronized as shown in the “box-car” timing diagram in Supplementary Figure 5. Time-resolved temperature measurements with millisecond resolution (Supplementary Figures 6, 7), in combination with gated thermal imaging, were used to record temperature fluctuations during individual pulses.

Two-dimensional (2-D) thermal images of the hot spots on both sides of the sample together with their positions relative to the spectrometer pinholes were recorded, displayed on viewing monitors (Figure 7d in the main text). The initially set lasers’ focus position and size were adjusted, in order to obtain precise alignment of the center of the hot spot with the spectrometer pinhole, the x-ray focus position was also aligned with the laser heating spot and the spectrometer pinhole (Figure 7c in the main text). The position of the sample with respect to laser was also adjusted, if necessary, in order to optimize the symmetry of the hot spots on both sides of the sample. After such initial adjustments, the position of the sample relative to the x-ray beam and spectrometer pinhole was kept constant throughout the remainder of the given experimental run; however, the focus size and position of the laser beams with the respect to the spectrometer pinhole may be adjusted if deemed necessary. In the majority of runs, at different pressure points, the laser power *v*. temperature relationship remained linear even up to temperatures significantly higher than the previously reported[2–5] Mo melting point temperatures (Supplementary Figure 7). The combination of short single-pulse heating and encapsulating the sample in a micro-fabricated single-crystal assembly[6] (Figure 7a,b in the main text) allowed to repeatedly reach previously unattainable and stable pressure and temperature conditions for Mo (Supplementary Figure 7).

Numerical model

Commercially available Comsol® 5.0 program and materials properties database were used to conduct numerical heat flow calculations.

In the model, a thin Mo sample disk is embedded into an MgO matrix, which acts as the pressure-transmitting medium and as a thermal insulating layer. The laser power is delivered onto both sides through the optically transparent MgO and couples with the Mo sample surface.

A thin platelet of molybdenum, encapsulated on all sides with optically transparent oxide layers (e.g. MgO, Al_2O_3), is placed and compressed between two opposing single-crystal diamond windows (i.e. anvils of the DAC). A rectangular pulse of radiant energy is introduced to the encapsulated sample through the diamond + oxide windows by two laser beams in a double sided LH-DAC arrangement[1]. Variations of the finite-element (FE) approaches have been used by others to obtain numerical solutions for either steady-state[8–12] or transient[13–15] temperature distributions in the laser-irradiated samples in this type of a DAC geometry. In the present study, we solve the time dependent heat equation:

$$\nabla \cdot (-k\nabla T) = Q - \rho C \frac{\partial T}{\partial t} \quad \text{(S1)}$$

where k, C, ρ, Q, T, and t denote thermal conductivity, heat capacity, density, heating power per unit volume (laser heat source), temperature, and time, respectively. The latent heat of melting/solidification is modeled with the apparent heat capacity approximation. Assuming that the phase transformation from solid to liquid occurs continuously over a finite temperature interval ΔT, the expression for C used in the heat equation may be formulated as

$$C = \frac{1}{\rho}\left[\theta \rho_{\mathrm{solid}} C_{\mathrm{solid}} + (1-\theta)\rho_{\mathrm{liquid}} C_{\mathrm{liquid}}\right] + C_{\mathrm{L}} \quad \text{(S2)}$$

where θ is a function representing the fraction of the solid. The C_{L} term in equation (S2) gives the distribution of the latent heat in the interval ΔT:

$$C_L(T) = L\frac{d\alpha_{\mathrm{m}}}{dT} \quad \text{(S3)}$$

where L and α_{m} denote the latent heat and mass fraction of the melt, respectively. Similarly, k and ρ are be formulated as

$$k = \theta k_{\mathrm{solid}} + (1-\theta)k_{\mathrm{liquid}} \quad \text{(S4)}$$

$$\rho = \theta \rho_{\mathrm{solid}} + (1-\theta)\rho_{\mathrm{liquid}} \quad \text{(S5)}$$

The heat equation is usually solved for the 3-dimensional DAC geometry using a 2-dimensional axisymmetric approximation. The set of boundary conditions listed in Supplementary Table 1 is used for in the calculations in the present study (Supplementary Figure 8). Temperature dependent values of the thermophysical properties of Mo, insulation layers, and diamonds were obtained from the materials database. A series of calculations were performed, wherein the boundary heat-source term, Q_{b}, was incrementally increased from zero until the substantial volume of liquid Mo was

found in the numerical solution. The calculations were performed assuming values of the thermophysical properties at ambient pressure for all the materials.

Sample temperature response

Our time-dependent heat flow model (see Numerical model section) indicated that the steady-state heating condition in a SMP heated LH DAC should be achieved after 100-200 μs after the switching on of the laser, and remain stable for the remainder of the square pulse. Indeed, stable heating, wherein the temperature response closely followed the modulation shape of the heating laser, was achieved when pulses were shorter than 5-20 ms. However, when heating to higher temperatures, ~2800 K and above, pulses longer than ~20 ms sometimes resulted in temperature instabilities and a runaway temperature response. Sudden temperature spikes sometimes happened, with an overshoot of the expected temperature by several thousand K for several milliseconds, followed by a decrease in temperature. Constraining of the heating pulse duration prevented the sudden run-away heating in most of our experimental runs. We note that continuous heating LH DAC, as was used in the past[2,4,5], is not a reliable approach for study of melting of molybdenum. Power levels, approaching the maximum 200-watt output of our laser heating system, were needed to reach required high *T* conditions. We also found that at such high laser power output, significantly longer time was needed to reach the steady state temperature conditions than was predicted by our heat flow model. The heat flow model used in this study, which assumes a constant temperature of the diamonds, is only approximately valid when very high heating laser power is used. We suspect that the application of very high laser power likely leads to significant heating the diamonds and the surrounding DAC components, which invalidates one of the model assumptions. In light of this, proper temperature state characterization our Mo melting experiments depended strongly on the use of time resolved thermometry.

Melt detection limit

Each solution, in the series of the calculations performed, was used to calculate the liquid phase distribution function $\theta_l(r, z)$ which quantifies the mass fraction of the liquid phase per unit volume in a specific LH DAC geometry. Due to the experimentally unavoidable thermal gradient, the volume of the melt is expected to be small, compared to the total volume of the sample. Therefore, in the quenched samples, the volume of the material quenched from a liquid state will be small compared to the total volume. In our experiments, the sample volume was probed by a micro-focused x-ray beam, normal to the flat sample surface and centered at $r = 0$. The radial intensity distribution of the micro focused x-ray beam was approximated by a Pseudo-Voight function $I_x(r)$, using experimentally constrained parameters[1]. The FWHM of $I_x(r)$ is comparable in size to the radial dimension of the volume of the material quenched from the liquid state; therefore, a significant fraction of the observed diffracted signal intensity is expected to originate from the un-molten, solid material. Consequently, a dimensionless parameter X_L was defined to quantify the fraction of the detected diffracted signal intensity originating from the material quenched from a liquid state.

$$X_L = \left[\iint_0^{r_s,z_s} \theta_l(r,z) \cdot I_x(r) drdz\right] / \left[\iint_0^{r_s,z_s} 1 \cdot I_x(r) drdz\right] \quad \text{(S6)}$$

where r_s and z_s denote the radius of the sample and the thickness of the sample, respectively. The numerically computed function $X_L(T)$ was used to estimate the errors melting temperature measurements, as described in the main text.

The results of the calculations led us to conclude that our experiment probe for melting detection is sufficiently sensitive for detecting the melt volume produced in the double-sided heating setup we used and should not result in significant T_M over-estimation, as we discussed in the main text. We note that single-sided laser heating approach, in contrast to the double-sided heating, produces a much smaller volume of melt (Supplementary Figure 9), and can lead to large T_M over-estimation error if used in combination with a bulk probe such as the XRD.

Additional sample notes

The majority of the data points correspond to a run done with a fresh sample. However, several of the reported data points correspond to runs done on samples that were used from in a previous run. The experimental pressures (P), and the obtained melting temperature data points (T_M), corresponding to the use of fresh vs. re-used sample are tabulated in Supplementary Table 2.

Demonstration of the proposed methodology on pure iron system

The quenched microstructure method for studying melting has been confirmed with pure iron (Fe) at a relatively low pressure of 36 GPa. Fe may be the most studied system in literature[16] for high pressure melting determination, displaying converging results at pressures below 50 GPa [16–21]. This makes the melting of Fe a suitable case for demonstration of the proposed methodology.

The study on Fe was carried out at a pressure of 36 (3) GPa. The sample loading was identical to that of Mo and single crystal MgO was used for thermal insulation. At this pressure and at room temperature Fe is a hexagonally-close-packed (HCP) metal (ε-Fe). Moreover, at this pressure, Fe is commonly expected to undergo a phase transition from hcp to a face-centered-cubic (FCC) polymorph (ɣ-Fe) at a temperature of 1500 (200) K ($T_{\varepsilon-\gamma}$).

Using the same approach as described for Mo, the ε-Fe microstructure showed incremental coarsening after each heating pulse at $T< T_{\varepsilon-\gamma}$ (Supplementary Figure 10a). The XRD of quenched Fe from temperature $T_{\varepsilon-\gamma}<T<T_M$ showed a mixture of coarse ɣ-Fe grains and ε-Fe (Supplementary Figure 10b). When heating Fe to a sufficiently high temperature, $T>T_M$ and quenching rapidly, fine-grained and continuous Debye rings were observed in the XRD images (Supplementary Figure 10c). The appearance of fine-grained Debye rings was accompanied by an abrupt weakening or disappearance of Bragg reflection spots from larger γ-Fe grains. The observed T_M of Fe [T = 2760(100) K] at a pressure of 36(3) GPa, observed in this study using the quenched microstructure approach, agees remarkably well with the majority of the most recent reports [16–21]. Supplementary Figure 11 shows the T_M and $T_{\varepsilon-\gamma}$, obtained using the quenched microstructure method of this study,

overlaid on Fe phase diagram from literature [17,18,20–22]. It is worthwhile to note that, in the case of Fe, the fine-grained microstructure after $T>T_M$ corresponds to the high-temperature phase, ɣ-Fe and not the ε-Fe. This gives an additional confirmation that the fine-grains, which we observe, is a high temperature phenomenon and corresponds to melting.

Supplementary References

1. Meng, Y., Hrubiak, R., Rod, E., Boehler, R. & Shen, G. New developments in laser-heated diamond anvil cell with in situ synchrotron x-ray diffraction at High Pressure Collaborative Access Team. *Rev. Sci. Instrum.* **86,** 72201 (2015).

2. Santamaría-Pérez, D. *et al.* X-ray diffraction measurements of Mo melting to 119 GPa and the high pressure phase diagram. *J. Chem. Phys.* **130,** 124509 (2009).

3. Yang, L., Karandikar, A. & Boehler, R. Flash heating in the diamond cell: melting curve of rhenium. *Rev. Sci. Instrum.* **83,** 63905 (2012).

4. Errandonea, D. *et al.* Systematics of transition-metal melting. *Phys. Rev. B* **63,** 132104 (2001).

5. Errandonea, D. Improving the understanding of the melting behaviour of Mo, Ta, and W at extreme pressures. *Phys. B* **357,** 356–364 (2005).

6. Hrubiak, R. *et al.* The laser micro-machining system for diamond anvil cell experiments and general precision machining applications at the High Pressure Collaborative Access Team. *Rev. Sci. Instrum.* **86,** 72202 (2015).

7. Du, Z., Amulele, G., Benedetti, L. R. & Lee, K. K. M. Mapping temperatures and temperature gradients during flash heating in a diamond-anvil cell. *Rev. Sci. Instrum.* **84,** 75111 (2013).

8. Bodea, S. & Jeanloz, R. Model calculations of the temperature distribution in the laser-heated diamond cell. *J. Appl. Phys.* **65,** 4688 (1989).

9. Dewaele, A., Fiquet, G. & Gillet, P. Temperature and pressure distribution in the laser-heated diamond--anvil cell. *Rev. Sci. Instrum.* **69,** 2421–2426 (1998).

10. Kiefer, B. & Duffy, T. S. Finite element simulations of the laser-heated diamond-anvil cell. *J. Appl. Phys.* **97,** 114902 (2005).

11. Konopkova, Z. *et al.* Thermal conductivity of hcp iron at high pressure and temperature. *High Press. Res.* **31,** 228–236 (2011).

12. Rainey, E. S. G., Hernlund, J. W. & Kavner, a. Temperature distributions in the laser-heated diamond anvil cell from 3-D numerical modeling. *J. Appl. Phys.* **114,** 204905 (2013).

13. Geballe, Z. M. & Jeanloz, R. Origin of temperature plateaus in laser-heated diamond anvil cell experiments. *J. Appl. Phys.* **111,** 123518 (2012).

14. Montoya, J. A. & Goncharov, A. F. Finite element calculations of the time dependent thermal fluxes in the laser-heated diamond anvil cell. *J. Appl. Phys.* **111,** (2012).

15. Morishima, H., Yusa, H. & Introduction, I. Numerical calculations of the temperature distribution and the cooling speed in the laser-heated diamond anvil cell. *J. Appl. Phys.* **83,** 4572–4577 (1998).

16. Saxena, S. & Eriksson, G. Thermodynamics of iron at high pressures and temperatures. *J. Phys. Chem. Solids* **84,** 271–279 (2015).

17. Alfè, D., Price, G. D. & Gillan, M. J. Iron under Earth's core conditions: Liquid-state thermodynamics and high-pressure melting curve from ab initio calculations. *Phys. Rev. B* **65,** 165118 (2002).

18. Anzellini, S., Dewaele, A., Mezouar, M., Loubeyre, P. & Morard, G. Melting of iron at Earth's inner core boundary based on fast X-ray diffraction. *Science.* **340,** 464 (2013).

19. Brown, J. M. & McQueen, R. G. Phase transitions, Grüneisen parameter, and elasticity for shocked iron between 77 GPa and 400 GPa. *J. Geophys. Res.* **91,** 7485 (1986).

20. Jackson, J. M. *et al.* Melting of compressed iron by monitoring atomic dynamics. *Earth Planet. Sci. Lett.* **362,** 143–150 (2013).

21. Shen, G., Prakapenka, V., Rivers, M. & Sutton, S. Structure of Liquid Iron at Pressures up to 58 GPa. *Phys. Rev. Lett.* **92,** 185701 (2004).

22. Aquilanti, G. *et al.* Melting of iron determined by X-ray absorption spectroscopy to 100 GPa. *Proc. Natl. Acad. Sci.* **112,** 12042–12045 (2015).
